# Heat Stroke

**Published:** 1903-03-28

**Authors:** 


### HEAT STROKE.

The subject of heat stroke is one of much interest to all those who, in the course of their professional career, may come to be placed in some portion of the globe where affections due to the heat of the sun's rays claim any considerable number of victims. Dr. Duncan, in a paper dealing with this question, and published in the *Edinburgh Medical Journal* this month, classifies the clinical varieties of heat stroke under three headings—(1) heat collapse; (2) direct heat stroke; (3) indirect heat stroke. A patient suffering from heat collapse suddenly becomes giddy and falls; the skin is moist and cool, the breathing hurried but not stertorous, the pulse small and soft, the pupils dilated, and the temperature at or below the normal; loss of consciousness is not as a rule complete, and recovery is usual. Of direct heat stroke or sunstroke proper there are several varieties. In one the victims are mostly unaccustomed to the fatigues of marching, and the affection is especially liable to occur when the air contains much moisture; violent headache is first complained of, and later the patient falls in convulsions, the teeth are clenched, insensibility is absolute, respiration is difficult, the pulse is small and irregular. In another the subject streams with sweat, becomes pale and cyanosed, with respiration shallow and quick; consciousness is not generally entirely lost, and revival occurs if the patient be laid down and relieved of all impediment to free respiration. In a third kind no fatigue is complained of, but the patient is thirsty and suddenly falls forward comatose; the state of coma may last 24 to 36 hours, and may end in death without recovery of consciousness. In a fourth variety, after a hot and wearying march in the sun the subject is seized with severe headache; this gradually becomes more intense, so that within twenty-four hours he may be rolling about in agony; soon great intolerance of light occurs, and after perhaps forty-eight hours unconsciousness sets in. Should the patient recover, the intense pain in the head may last six or eight weeks, and then it gradually abates. The commonest form of heat stroke is the indirect form. Here the patient is usually attacked after retiring into a hot and close atmosphere. At the onset the skin is pale; there is nausea, colic, and incontinence of urine; convulsions follow, to be succeeded by cyanosis, dyspnœa, and insensibility; the breathing is stertorous, and the temperature of the body may reach 108° or 110°. The affection is more liable to occur among new arrivals in a hot climate than among old residents, and hot days in the cooler season of the year are especially dangerous. The plethoric and intemperate, those suffering from fatty heart, or who have had syphilis are found to be particularly predisposed. Alcohol can destroy the comparative immunity possessed by the coloured races. In this connection it has been shown in America that the negro, when he removes to the cities and drinks to excess, becomes as liable to heat stroke as the white races. Overcrowding is a dangerous factor, both in the open and under cover; and marching in close order is to be avoided in a tropical climate. Moist air, absence of wind, and hot winds are all predisposing causes. Alcoholic drinks should also be eschewed where there is exposure to a hot sun. As to dress, the head covering should be made from some substance which is a bad conductor of heat without being unduly heavy. Old European residents in Egypt, whenever they make an expedition into the desert, are accustomed to wear a tight jean skull-cap, similar to that worn by Arabs under the turban, next to the head, and experience seems to have quite established the efficacy of this method. It is most important to protect the eyes by the use of neutral-tinted glasses; and, finally, the spinal cord must be protected by a thick woollen pad sewn into the coat. The dress must be loose round the neck, chest, and abdomen, and the material should be a light woollen one. Dr. Duncan himself suffered severely and repeatedly from the effects of the sun until he adopted the plan of lining his helmet and coat with yellow, after which, although exposed for some years to extreme heat, he had no further attacks. He therefore strongly recommends similar measures to be made use of by all those who cannot withstand the direct heat of the sun. With regard to treatment during an attack, the patient should be moved into the shade where possible, his clothes unfastened, and cold water poured upon his head and neck; ammonia also should be applied to his nostrils. The douche must be repeated until a favourable effect is produced. If convulsions occur, a few whiffs of chloroform are indicated. Venesection should not be performed.

